# Socioeconomic determinants of unmet need for outpatient healthcare services in Iran: a national cross-sectional study

**DOI:** 10.1186/s12889-021-10477-6

**Published:** 2021-03-06

**Authors:** Sajad Vahedi, Amin Torabipour, Amirhossein Takian, Saeed Mohammadpur, Alireza Olyaeemanesh, Mohammad Mehdi Kiani, Efat Mohamadi

**Affiliations:** 1 Department of Health Services Management, School of Public Health, Ahvaz Jundishapur University of Medical Sciences, Ahvaz, Iran; 2 Social Determinants of Health Research Center, Ahvaz Jundishapur University of Medical Sciences, Ahvaz, Iran; 3 Health Equity Research Centre (HERC), Tehran University of Medical Sciences, Tehran, Iran; 4 Department of Global Health & Public Policy, School of Public Health, Tehran University of Medical Sciences, 2nd Floor, Main Building, Poursina Ave, Tehran, Iran; 5 Department of Health Management, Policy & Economics, School of Public Health, Tehran University of Medical Sciences (TUMS), 2nd Floor, Main Building, Poursina Ave, Tehran, Iran; 6 Department of Health Economics, School of Management and Medical Information, Iran University of Medical Sciences, Tehran, Iran; 7 National Institute of Health Research (NIHR), Tehran University of Medical Sciences, Tehran, Iran

**Keywords:** Iran, Healthcare services, Access, Unmet need

## Abstract

**Background:**

Unmet need is a critical indicator of access to healthcare services. Despite concrete evidence about unmet need in Iran’s health system, no recent evidence of this negative outcome is available. This study aimed to measure the subjective unmet need (SUN), the factors associated with it and various reasons behind it in Iran.

**Methods:**

We used the data of 13,005 respondents over the age of 15 from the Iranian Utilization of Healthcare Services Survey in 2016. SUN was defined as citizens whose needs were not sought through formal healthcare services, while they did not show a history of self-medication. The reasons for SUN were categorized into availability, accessibility, responsibility and acceptability of the health system. The multivariable logistic regression was used to determine significant predictors of SUN and associated major reasons.

**Results:**

About 17% of the respondents (*N* = 2217) had unmet need for outpatient services. Nearly 40% of the respondents chose only accessibility, 4% selected only availability, 78% chose only responsibility, and 13% selected only acceptability as the main reasons for their unmet need. Higher outpatient needs was the only factor that significantly increased SUN, responsibility-related SUN and acceptability-related SUN. Low education was associated with higher SUN and responsibility-related SUN, while it could also reduce acceptability-related SUN. While SUN and responsibility-related SUN were prevalent among lower economic quintiles, having a complementary insurance was associated with decreased SUN and responsibility-related SUN. The people with basic insurance had lower chances to face with responsibility-related SUN, while employed individuals were at risk to experience SUN. Although the middle-aged group had higher odds to experience SUN, the responsibility-related SUN were prevalent among elderly, while higher age groups had significant chance to be exposed to acceptability-related SUN.

**Conclusion:**

It seems that Iran is still suffering from unmet need for outpatient services, most of which emerges from its health system performance. The majority of the unmet health needs could be addressed through improving financial as well as organizational policies. Special attention is needed to address the unmet need among individuals with poor health status.

## Background

As one of the most fundamental dimensions of social justice, access to healthcare services is often considered as a pillar of equity in any healthcare system [1, 2]. Evaluating access to required healthcare services is the key factor for healthcare policy makers to address inequalities in the healthcare delivery and boost health outcomes [3, 4]. However, access is a relatively complex and multidimensional concept that could not be directly measured [5]. The literature emphasizes on unmet healthcare need as a critical indicator of access to healthcare services [6]. This measure provides insights into the barriers faced by people when they seek medical care, which can in turn reflect financial, physical, and cultural accessibility of healthcare services [7]. Unmet need could be defined as ‘the differences between services judged necessary to deal appropriately with health problems and services actually received’ [8]. Hence, the need is considered unmet if individuals could not receive required treatment that was believed to improve their health condition.

Two main approaches exist to measure unmet need for healthcare services: clinical and subjective [8]. In the clinical approach, which in its optimistic manner is based on clinical guidelines, judgments of healthcare professionals are used to determine whether the person has received appropriate care or not [6]. While this approach could lead to the underestimation of the wider concept of unmet need, it seems to well operate only in smaller scales such as a clinic or a hospital. In the second approach, a subjective evaluation of the individuals’ healthcare needs and whether they have received necessary care during the specified time period are used to determine unmet need [9]. Given the wider concept of need and also the possibility of implementing healthcare surveys in widespread settings [6], previous studies have used subjective measurement of unmet need as an easy way to estimate access to required healthcare services within various settings.

Unmet need for healthcare services could be also categorized according to its different causes such as accessibility, availability and acceptability of the services [10]. Unmet need due to accessibility is related to financial barriers to access required services [6]. Availability refers to unmet need related to the organizational aspect of the healthcare system such as waiting lists, services unavailable when required, and services unavailable in an area [9]. Unmet need due to acceptability could be emerged when individuals are not able to take time off work or cultural conditions [11]. While availability and accessibility of healthcare services are related to the health system performance, acceptability of the services is defined by expectations and circumstances of clients of the health system [6, 9, 11].

Most of research evidence on unmet need for healthcare services comes from high-income countries in North America [12, 13], Europe [6, 9, 14], and Asia [11, 15]. The studies revealed that the level of unmet need and accessibility-related unmet need were negligible in the Western European countries and Canada. In contrast, South Korea and Serbia showed higher levels of unmet need due to accessibility of healthcare services. In Iran, a previous study [16] reported huge unmet need. Nonetheless, the study did not differentiate among accessibility, availability and acceptability of unmet need. The main objectives of the current research were to create new evidence on the level of unmet need and its reasons, as well as to determine various predictors of this unwanted outcome and its reasons in the Iranian health system.

## Methods

This is a cross-sectional study. We conducted a secondary analysis of the data obtained from the Iranian Healthcare Utilization Survey (IrUHS) of 2016. The IrUHS included all individuals in both ordinary and group households living in urban and rural areas of Iran. The planned sample size in this survey, which was obtained through three-stage cluster sampling, was 22,470 households comprising 76,674 individuals. Sampling units were based on the national census of the Iranian Statistical Centre in 2011. All members of the selected households were interviewed and finally 74,857 individuals responded to the survey (response rate: 97.6%).

IrUHS consisted of two questionnaires entitled Household Questionnaire (to collect household socio-demographic and healthcare needs information) and Individual Questionnaire (to gather detailed information of healthcare utilization). The data collection was based on face-to-face interviews with the surveyed individuals [17]. The participants were asked about their outpatient and inpatient healthcare needs in the household questionnaire. Respectively, 2 weeks and 12 months preceding the interviews were considered as the recall period for outpatient and inpatient healthcare needs. The participants with a history of healthcare needs were asked about the utilization of healthcare services in the individual questionnaire. This history for outpatient healthcare need was obtained from the following question: Was there any time during the past 2 weeks that you experienced any need for outpatient care? The respondents older than 15 years who had outpatient healthcare needs (13,005 individuals) were included in this research.

### Measuring subjective unmet need and its reasons

Generally, there are two main approaches to measure subjective unmet need (SUN). Some studies used a general survey questionnaire to measure the extent and potential causes of self-perceived unmet healthcare needs [9, 13, 18–20]. In the second approach, survey data was used to identify those with a need for healthcare, followed with subsequent examination of their utilization of required healthcare services [6, 16, 18, 21]. Due to lack of a general survey containing specific questions about unmet healthcare need in Iran, we adopted the second approach to measure SUN in this research. This was indirectly defined by the following question: “Was there any time during the past two weeks that you utilized outpatient healthcare services?” Two groups of respondents were formed; those who used healthcare services and those who did not. In the latter group, those who had self-medication were considered as the individuals with met healthcare needs and were added to the former. Hence, the individuals with SUN were considered those who did not use formal healthcare services and did not have a history of self-medication in the last 2 weeks.

Those who did not utilize healthcare services were asked about the reasons for not using the services. This was performed through ten questions in the individual questionnaires. According to the previous literature on the reasons for unmet healthcare, the answers to these questions were categorized into three main reasons for unmet healthcare needs. The first group of responses decreased the accessibility of health care services through lower affordability (Accessibility). The second group of responses dealt with the waiting list and availability of healthcare services (Availability). Similar to previous studies [6, 22], the unmet need due to accessibility and availability accounted for the responsibility of the health system. The third group was about the personal circumstances of the responders such as the postponement of healthcare needs due to lack of free time or other circumstances (Acceptability). The definition of unmet need and its different reasons could be found in Table 1.

**Table 1:** Definition of overall unmet need and its different reasons

Overall unmet need	Required healthcare needs which are not met
Unmet need due to accessibility	Required healthcare needs which are not met, because there are insufficient resources
Unmet need due to availability	Required healthcare needs which are not met, because the required services are not available
System-related unmet need	Required healthcare needs which are not met, because of accessibility and availability of healthcare services
Unmet need due to acceptability	Required healthcare needs which are not met because of the postponement of healthcare needs due to lack of free time

### Variables

Andersen’s Behavioral Model of Health Services Use was used to explore potential determinants of SUN. This model assumes that utilization of healthcare services is a function of predisposing, enabling and need factors [23, 24]. According to the model, predisposing factors include demographic characteristics such as age, sex, marital status and family size, plus social structure such as employment, education and ethnicity. Moreover, material resources such as income, health insurance and distance from healthcare services were considered as enabling factors. Severity of illness, self-rated health and multiple chronic conditions were also considered as the need factors in this model [24].

Considering Anderson’s model, predisposing factors included in this study were sex (male/female), age (< 30, 30–59, and ≥ 60 years), marital status (married/unmarried), educational status (illiterate, primary, secondary, and diploma or higher), and employment status (employed/unemployed). On the other hand, area of residence (urban/rural), economic status (poorest, poor, middle, rich and richest), and health insurance (basic and complementary) were included as enabling factors. Furthermore, the number of outpatient healthcare needs (one/two or more) was used as a need factor.

### Statistical analysis

The principal component analysis was used to create economic status by using asset data such as having a separate kitchen, central heating, telephone usage, computer, Internet access at home, owning a motorcycle or a car, and whether the person owned a house or not. This statistical scheme has been widely used in previous studies [23, 25–27]. The Pearson’s chi-square tests was also used to analyze the differences between the respondents with unmet and met healthcare needs. A logistic regression analysis with maximum likelihood was used to analyze the determinants of SUN and their major reasons. We also calculated the odds ratios (OR) and 95% confidence intervals (CIs). The Hosmer-Lemeshow test was used to goodness of fit. According to this test, a good model fit is indicated by a value of *P* > .05. The results were considered statistically significant when the *p*-value was < 0.05. The analysis was performed by using the Stata/SE version 12.0.

## Results

### Descriptive results

While 17.05% of the respondents aged 15 and older had unmet need for outpatient healthcare services, 82.95% of them met their healthcare needs through formal care or self-medication. The characteristics of the population reporting unmet and met healthcare needs are presented in Table 2. There was a statistically significant difference between the individuals with met and unmet healthcare needs with regard to different variables. Although there was no difference between male and female participants in terms of unmet and met healthcare needs, different frequencies were observed among various age groups. The highest percentage of the individuals with unmet healthcare needs was in the age group of 30–59 years.

**Table 2:** Characteristics of the population reporting unmet and met healthcare needs in Iran 2016

	Unmet healthcare needs	Met healthcare needs	*p*-value^*^
	2217 (17.05)	10,788 (82.95)	
**Predisposing factors**			
Sex:			0.156
Male	918 (17.61)	4292 (82.39)	
Female	1299 (16.66)	6496 (83.34)	
Age:			< 0.001
Under 30	433 (14.95)	2462 (85.05)	
30–59	1282 (18.13)	5786 (81.87)	
60 and above	502 (16.50)	2540 (83.50)	
Marital status:			0.030
Married	1659 (17.06)	8061 (82.94)	
Widowed or divorced	262 (19.02)	1115 (80.98)	
Single	296 (15.51)	1612 (84.49)	
Education:			< 0.001
Illiterate	700 (20.31)	2746 (79.69)	
Primary	636 (18.96)	2717 (81.87)	
Secondary	336 (15.96)	1768 (84.04)	
Diploma or higher	545 (13.28)	3557 (86.72)	
Employment status:			0.097
Employed	559 (16.74)	2542 (83.26)	
Unemployed	1658 (18.02)	8246 (81.98)	
**Enabling factors**			
Area of residence:			< 0.001
Urban	1349 (15.50)	7349 (84.50)	
Rural	868 (20.15)	3439 (79.85)	
Economic status:			< 0.001
Poorest	622 (23.65)	2008 (76.35)	
Poor	458 (17.73)	2125 (82.27)	
Middle	438 (16.90)	2153 (83.1)	
Rich	397 (15.26)	2203 (84.74)	
Richest	302 (11.61)	2299 (88.39)	
Basic health insurance:			0.064
Yes	2063 (16.89)	10,150 (83.11)	
No	154 (19.44)	638 (80.56)	
Complementary health insurance:			< 0.001
Yes	281 (11.44)	2174 (88.56)	
No	1936 (18.35)	8614 (81.65)	
**Need factors**			
Number of outpatient needs:			< 0.001
One	1451 (13.92)	8969 (86.08)	
Two or above	766 (29.63)	1819 (70.37)	

*p-value: Chi-square test on the difference of unmet and met needs of health care across different socio-demographic groups

Regarding marital status, widowed or divorced individuals and single ones had respectively the highest and lowest prevalence of unmet healthcare needs. While significant differences were observed among the individuals of different education levels, there was no significant difference between the employed and unemployed persons. The largest percentages of the respondents with unmet healthcare needs were living in rural areas. With increasing economic quintiles, the frequency of unmet healthcare needs for healthcare services was decreasing, until the greatest unmet need was found in the poorest quintile. The respondents that had health insurance (basic and complementary) showed significantly lower unmet healthcare needs. The highest unmet healthcare needs were observed among the individuals with two or more outpatient needs.

Table 3 presents the main reasons that the respondents expressed for their unmet healthcare needs. While about 40.32% of the individuals chose only accessibility, acceptability (12.76%) and availability (3.65%) were solely considered as the other main causes of unmet healthcare needs. Furthermore, the majority of individuals (78.35%) simultaneously chose accessibility and availability as the main reasons.

**Table 3:** The main reasons why respondents did not meet their healthcare needs, Iran 2016

Reason why respondent did meet their healthcare needs	N	%
Only Accessibility	894	40.32
Only Availability	81	3.65
Accessibility and Availability (Responsibility of healthcare system)	1737	78.35
Only Acceptability	283	12.76
No response	197	8.88

### Socioeconomic determinants of unmet healthcare need

Three models of multivariable logistic regression were used to show the association of predisposing, enabling and need factors with unmet healthcare needs (Table 4). There was no significant association between sex and SUN, as well as the SUN arisen from responsibility or acceptability of the healthcare system. While the middle- age group was at a higher risk to face with SUN (1.19 [1.00–1.42]), older individuals had lower odds to experiencing SUN due to the responsibility of the healthcare system (0.71 [0.55–0.92]). Furthermore, there was significant association between the higher-age groups and acceptability-related SUN. We found no significant relationship between marital status and SUN in the three estimated regression models. Although having a lower education level significantly increased the likelihood of SUN and responsibility-related SUN, individuals with lower levels of education had a lower chance to experience acceptability-related SUN. Employed subjects had a greater chance of facing with unmet need in all the regression models, but this was only significant for SUN (1.18 [1.01–1.36]).

**Table 4:** Multivariable logistic regression models for SUN^a^ and its different categories of reasons, Iran 2016

	SUN		Responsibility-related SUN		Acceptability-related SUN	
	OR (95% CI)	*p*-value	OR (95% CI)	*p*-value	OR (95% CI)	*p*-value
**Sex**						
Male	1		1		1	
Female	0.90 (0.78–1.02)	0.109	0.89 (0.77–1.03)	0.114	1.02 (0.73–1.44)	0.897
**Age**						
Under 30	1		1		1	
30–59	1.19 (1.00–1.42)	0.047	1.13 (0.93–1.36)	0.225	1.61 (1.03–2.51)	0.035
60 and above	0.84 (0.67–1.05)	0.130	0.71 (0.55–0.92)	0.009	1.77 (1.03–3.05)	0.039
**Marital status**						
Married	0.86 (0.72–1.04)	0.129	0.87 (0.71–1.07)	0.197	1.24 (0.77–2.00)	0.380
Widowed or divorced	0.89 (0.69–1.16)	0.408	0.86 (0.64–1.16)	0.319	1.57 (0.85–2.90)	0.151
Single	1		1		1	
**Education**						
Illiterate	1.23 (1.02–1.48)	0.034	1.56 (1.26–1.93)	< 0.001	0.49 (0.31–0.76)	0.002
Primary	1.18 (1.00–1.39)	0.045	1.46 (1.21–1.76)	< 0.001	0.61 (0.35–0.74)	< 0.001
Secondary	1.00 (0.84–1.20)	0.962	1.19 (0.97–1.46)	0.098	0.70 (0.39–0.95)	0.028
Diploma or higher	1		1		1	
**Employment status**						
Employed	1.18 (1.01–1.36)	0.032	1.17 (0.99–1.37)	0.062	1.42 (0.99–2.04)	0.058
Unemployed	1		1		1	
**Area of residence**						
Urban	1		1		1	
Rural	1.08 (0.96–1.21)	0.196	1.07 (0.94–1.21)	0.288	1.17 (0.86–1.59)	0.311
**Economic status**						
Poorest	1.88 (1.54–2.29)	< 0.001	2.35 (1.87–2.96)	< 0.001	0.77 (0.43–1.38)	0.373
Poor	1.42 (1.16–1.73)	< 0.001	1.72 (1.36–2.18)	< 0.001	0.88 (0.66–1.37)	0.625
Middle	1.38 (1.14–1.68)	< 0.001	1.76 (1.39–2.22)	< 0.001	0.82 (0.53–1.26)	0.365
Rich	1.34 (1.11–1.63)	< 0.001	1.53 (1.21–1.93)	< 0.001	1.23 (0.80–1.90)	0.336
Richest	1		1		1	1
**Basic health insurance**						
Yes	0.82 (0.65–1.02)	0.078	0.76 (0.60–0.97)	0.026	1.14 (0.67–1.93)	0.350
No	1		1		1	
**Complementary health insurance**						
Yes	0.75 (0.63–0.89)	< 0.001	0.60 (0.49–0.74)	< 0.001	1.51 (0.64–1.56)	0.350
No	1		1		1	
**Number of outpatient needs**						
One	1		1		1	1
Two or above	2.58 (2.29–2.90)	< 0.001	3.06 (2.69–3.47)	< 0.001	1.54 (1.10–2.16)	0.013
Hosmer-Lemeshow goodness-of-fit test	Chi-Square = 8.39, *p*-value = 0.396		Chi-Square = 12.40, *p*-value = 0.134		Chi-Square = 10.06, *p*-value = 0.261	

^a^ Subjective Unmet Need

*p* < 0.05

*C.I.* Confidence Intervals

There was no significant association between area of residence and SUN and their different reasons. Economic status was found to be associated with SUN and responsibility-related SUN. In this regard, the poorest quintile presented the largest odds for SUN (1.88 [1.54–2.29]) and responsibility-related SUN (2.35 [1.87–2.96]). In terms of health insurance, individuals with basic health insurance had a lower chance of facing with SUN due to the responsibility of the healthcare system (0.76 [0.60–0.97]), while those who had complementary insurance showed a lower chance of SUN in the first (0.75 [0.63–0.89]) and second (0.60 [0.49–0.74]) models. The number of outpatient needs, as a need factor, was only significant predictor of SUN in the three estimated regression models. The individuals with two or more outpatient healthcare needs had a higher odd to experience SUN (2.58 [2.29–2.90]), SUN due to the responsibility of the healthcare system (3.06 [2.69–3.47]) and acceptability-related SUN (1.54 [1.10–2.16]).

## Discussion

This study aimed to measure unmet need for outpatient healthcare services and to explain the factors associated with it and its different reasons in Iran. Our research indicated that 17.05% of those who had outpatient healthcare needs could not access to required healthcare services or ignored self-medication to meet their healthcare needs. A previous study conducted in Iran [16] showed that almost 36% of individuals in need did not seek any outpatient healthcare services, which is far from our findings. It seems that former research did not adjust unmet need for self-medication. According to both definitions, the prevalence of unmet need in Iran is much higher than it was reported in the previous studies in Europe [6, 9, 28]. Let alone, some studies in South Korea [11, 15] reported a higher rate of unmet need in comparison to those in our research. As need concept and accordingly SUN is a normative issue as well as a multi-dimensional outcome [18], the discrepancy between different studies could be justifiable. Compared to most previous studies that reported annual prevalence, we reported biweekly SUN for outpatient healthcare services that could provide a more precise picture of this unwanted outcome.

Taking into consideration the reasons behind unmet healthcare need could help decrease its occurrence [18]. We observed that accessibility solely related to nearly 40% of the overall unmet need for outpatient healthcare services. In addition, while about 4% of the studied people forgot their needs due to the availability of healthcare services, most of them collectively experienced unmet need due to accessibility and availability of healthcare services that was highly related to the organization and financing of the healthcare system. Hence, the need for appropriate and evidence-based policies in both healthcare organization and financing is clear to decrease most of unmet need in Iran. We advocate more attention to facilitate access to the outpatient sector within the ongoing Health Transformation Plan to decrease unmet need in Iran [29]. On the other hand, only about 12% of observed unmet need was categorized as unmet need due to acceptability of healthcare services. This kind of unmet need is strongly related to the expectations and circumstances of the clients, which is likely to be decreased through enhancing health literacy [30]. Previous studies carried out in other settings reported different reasons for unmet need. While in some European countries [20] and Canada [31] acceptability or availability of healthcare services caused unmet need, in Serbia [6] and South Korea [11], like our finding, unmet need due to accessibility were prevalent.

Our study revealed that poor health status was the only significant factor that increased unmet need in the three estimated models. This was in line with former studies [9, 11, 12, 14] that showed worsen health status could increase unmet need for healthcare services. Poor health status was the strongest predictors of unmet need in the first and second regression models. Compared with the individuals who had one outpatient need, those with two or more outpatient needs were at a higher risk of facing with overall, system-related unmet need and unmet need due to acceptability of healthcare services. This indicates that the Iranian healthcare system might presumably suffer from high degrees of inequity, at least within its outpatient sector [27]. We strongly recommended provision of healthcare services for those with higher needs, which could in turn lead to improving vertical and horizontal equity in Iran.

Education was the other significant factor that modified unmet need in the three estimated models. While less educated individuals suffered from unmet need and those caused by the performance of the health system, they showed, rather unexpectedly, a lower chance to expose unmet need due to accessibility. Less educated people suffered from financial strains that made them vulnerable to unmet need due to the performance of the health system. Nevertheless, their needs were not overlooked due to healthcare services acceptability. This might be associated with lower expectations of the health system among this group. This finding is inconsistent with the results of previous studies in Iran [16] and Europe [9], in which no significant association was found between education and unmet need, and the studies carried out in Canada [12, 22] that showed higher education could increase unmet need. Nonetheless, a large number of previous studies [6, 11, 20, 32] affirmed the positive association between lower education and overall and system-related unmet need. In the present study, gender and marital status had no significant association with unmet need and their different causes. A recent study in Iran also found no significant relationship between these factors and outpatient healthcare utilization [33].

We found that employed people were in a higher risk of facing with overall unmet need and the ones caused by the responsibility of the health system, which is in line with the previous studies [6, 11]. Employed people may neglect their needs to bring enough affordability for their dependents. We observed different effects of age groups on unmet needs in Iran. First, in contrast with previous studies [6, 9, 11], the middle aged group had a higher chance to face with SUN. One explanation for this finding could be the Iranian parents ignoring or at least not prioritizing the healthcare needs of themselves to meet the needs of their dependents. Let alone that many individuals in this age group were employed people, whose higher unmet need is expected. Former study in Iran also showed that the middle-age and employed people had decreased rates of healthcare utilization, compared with their peers [33]. We advocate paying greater attention to the healthcare needs among middle-age individuals to guarantee healthier labor force. Second, the elderly had a lower odd to experience unmet needs. This could be the result of prioritizing elderly citizens as a target group by the Iranian health system [6, 9]. Iran has a relatively comprehensive pension schemes for retired citizens [34], which seems that covers most healthcare expenditures for the elderly. We recommend the expansion of basic health insurance in Iran [27, 34] to increase the access to required healthcare services and decrease the responsibility-related SUN among older Iranians. Third, we observed that aging could increase the acceptability-related SUN. While acceptability-related SUN were less prevalent among higher age groups in Europe and Canada [9, 12], other study in South Korea [11] found no significant association between acceptability-related SUN and age. It seems that opportunity cost of devoting enough time for healthcare seeking among middle-age and older adults is high in Iran, which might be related to the economic depression.

As far as enabling factors were concerned, our research revealed that lower economic status was accompanied with higher odds of overall unmet need and unmet need due to performance of health system. This might indicate that poor individuals predominately suffered from unmet need due to the health system responsibility. In line with our finding, other studies [9, 11, 14, 16, 21] also showed that lower economic status was associated with a higher degree of overall unmet need as well as system-related unmet need. Poor people not only had lower affordability but also might live in areas with worse access to healthcare services, which might in turn make them vulnerable to unmet need. Our study also showed that having complementary insurance could not only decrease the probability of overall unmet need, but also bring down unmet need related to the health system. The odds ratio of basic health insurance was significant only in the second model. Further, we observed that basic health insurance significantly decreased only unmet need due to the responsibility of the health system. As complementary health insurance might not be accessible to disadvantaged groups, policy makers need to do their utmost efforts to create inclusive basic insurance programs to tackle unmet need effectively. Previous studies [32, 35] also confirmed the protective effect of health insurance against unmet need.

### Rigor of study

Despite providing valuable evidence on the feature of unmet need in Iran, this study had some limitations that need to be acknowledged. First, we used the IrUHS to measure the unmet need related to healthcare services. This survey was intrinsically designed to study the utilization of healthcare services and did not have any questions about clinical conditions, such as history of chronic disease or activity daily living that could decrease comparability of our results with those of previous studies. However, it provided an opportunity to estimate unmet need in the 2 weeks prior to the survey that could reduce recall bias. It is recommended that future studies to use specific questionnaire with wide-range questions about health status in Iran, which will help more effective measurement of unmet healthcare needs in Iran. Second, this cross-sectional study could not necessarily bring any causality association between different predictors and unmet need, which is required to be studied in the long-term. Third, this study did not use supply-side variables such as provider’s characteristics that might modify the unmet need. Finally, we only studied the unmet need in the outpatient sector that could not reflect a complete picture of such an unwanted outcome in the entire health system of Iran. Hence, the status of unmet need in the inpatient sector, especially after HTP, needs to be addressed in future studies. Nevertheless, this study brought new evidence on the main causes of unmet healthcare needs and provided policy implications to tackle this negative outcome in the outpatient health sector in Iran.

## Conclusions

Exploring unmet healthcare needs could assist policy makers to evaluate access to healthcare services. This study revealed that Iran is still suffering from unmet need in its outpatient sector. Less educated individuals were at a higher risk to experience overall unmet need as well as system-related unmet need, whereas they showed a lower chance of facing with acceptability-related unmet need, simultaneously. While overall and system-related unmet need was prevalent among employed individuals and disadvantaged groups, both basic and complementary health insurances might provide protection against unmet need. As poor health status was a major determinant of outpatient unmet need, greater attention is required to reduce inequity in the health system of Iran that is predominantly arising from the accessibility of healthcare services. Since the country has been gearing up towards the implementation of Sustainable Development Goals, particularly Universal Health Coverage (UHC) by 2030, more emphasis on system-related policies such as improvement of the financing and organization of healthcare services are essential to reduce most of the unmet need and bridge the UHC gap in Iran.

## Data Availability

The data that support the findings of this study are available from the National Institute of Health Research (NIHR) in Iran but restrictions apply to the availability of these data, which were used under license for the current study, and so are not publicly available. Data are however available from the authors upon reasonable request and with permission of NIHR.

## Abbreviations

SUN: Subjective Unmet Need
IrUHS: Iranian Healthcare Utilization Survey
UHC: Universal Health Coverage
